# Paliperidone long-acting injection in the treatment of an adolescent with schizophrenia with fluctuating mental symptoms during menstrual period: a case report

**DOI:** 10.3389/fpsyt.2023.1276983

**Published:** 2023-10-06

**Authors:** Feng Wang, Juan Chen, Linglin Guo, Zhixiong Li, Zhe Li

**Affiliations:** ^1^Mental Health Center, West China Hospital, Sichuan University, Chengdu, China; ^2^West China School of Nursing, Sichuan University, Chengdu, China; ^3^The Third Department of Clinical Psychology, Karamay Municipal People’s Hospital, Karamay, China; ^4^Sichuan Clinical Medical Research Center for Mental Disorders, Chengdu, China

**Keywords:** paliperidone extended-release tablets, paliperidone palmitate injection, schizophrenia, menstrual cycle, adolescent

## Abstract

**Background:**

The treatment of schizophrenia, a chronic disabling psychiatric disorder, mainly relies on antipsychotics to control the disease and improve clinical symptoms. Various medication options are available, and differences in treatment effects, even for the same medication, have been noted. Treatment efficacy is correlated with the patient’s sex, age, and physical condition. When a drug fails to achieve the desired effect or the symptoms are unstable, the drug dose is often increased or a change in medication is advised according to the patient’s situation.

**Case presentation:**

We report the case of a 16 years-old girl with schizophrenia and apparent psychotic symptoms. According to the genetic testing results, the symptoms were effectively controlled, and she was discharged from the hospital with the prescription of paliperidone sustained-release tablets. During the follow-up, her symptoms fluctuated during menstrual period, causing her great distress. Furthermore, her compliance gradually declined during the following 2 years of treatment, and the medication was often discontinued. We changed the drug from an oral tablet to an injection preparation while maintaining the active ingredients of the drug. The patient’s symptoms were significantly controlled, and no fluctuation of symptoms occurred during the menstrual cycle.

**Conclusion:**

Long-acting antipsychotic injections can be administered to female adolescents with schizophrenia who experience fluctuating psychotic symptoms during menstruation. This technique can ensure both consistency of medication and improvement in clinical symptoms.

## Background

The long-term maintenance treatment of schizophrenia mainly relies on drugs, including oral preparations and intramuscular injections; most patients are treated with oral drugs. However, the efficacy of oral drug therapy may be influenced by various factors. Clinicians usually adjust the dose or type of oral medication according to the patient’s condition (1). The range of antipsychotic drugs available is limited. Thus, before switching medications, clinicians should consider many factors, including the patient’s tolerance to the drug, absorption and metabolic rates, and the patient’s hormone levels. However, changes in hormone levels in women during clinical treatment are usually an easily overlooked factor.

As the pathogenesis of schizophrenia is not fully understood, current antipsychotics have certain limitations (2). Symptoms fluctuating with the menstrual cycle have been widely reported in women with schizophrenia; the occurrence of premenstrual psychosis and recurrence of psychotic features despite adherence to antipsychotic medications at the onset of or during menstrual cycle in adolescent girls and young women have been reported (3). Furthermore, symptoms in patients with schizophrenia reportedly change at different stages of the menstrual cycle (premenstrual, menstrual, and post-menstrual). Therefore, targeted treatment measures must be applied in such cases (4). For patients with poor treatment effectiveness or with disease fluctuation, clinicians usually increase the dose gradually, change the type of drugs, or administer combination drugs according to the patient’s mental symptoms, physical condition, compliance, and drug interactions, and constantly adjust the treatment plan to select the appropriate antipsychotic drugs. Switching medications requires re-titration, which may cause fluctuating symptoms. Furthermore, dose escalation or combination therapy may also reduce patient adherence to therapy. Additionally, it is necessary to weigh the advantages and disadvantages of changing drugs to maximize patient benefits. Another option would be changing the form of the same drug; however, the effectiveness of this strategy in female patients with fluctuating mental symptoms during menstruation has not been reported.

Here, we report the case of a 16 years-old girl with schizophrenia who presented with recurrent psychotic symptoms during the menstrual cycle and was managed using a long-acting injection of the same drug.

## Case presentation

### Chief complaints

A 16 years-old female patient was admitted to the hospital in September 2018. Six months before admission, the patient had been depressed after an argument with classmates at school and had frequent dizziness, slowness, and inattention at home. She could hear someone scolding her out of thin air, felt that her stomach is “talking” and will spread her ideas to her classmates, suspected that her classmates were discussing her affairs, and needed to repeatedly ask others to ensure that her classmates did not hear her swear words. Gradually, she became inattentive, talked to herself, did not take the initiative to communicate with classmates or family members, often felt nervous and afraid, slept poorly at night, and her academic performance decreased significantly.

### Personal history and family history

Since childhood, she had been introverted, sensitive, cared about others’ opinions, and had almost no interests. Her parents divorced 4 years ago, and she lived with her mother; however, the patient had good support from family members.

### History of past illness

The patient was in good health, had never smoked or consumed alcohol, and had no family history of mental disorders. She did not report any major adverse life events. Both the patient and her family denied any substance abuse. She had no history of violent, agitated, or suicidal behavior during her illness.

### Physical examination and laboratory examinations

Routine blood tests did not reveal any unusual findings. No abnormalities were found in liver, kidney, and metabolic function; coagulation tests; transfusion; immunity; routine urine examination; and procalcitonin and adrenocorticotropic hormone levels. Magnetic resonance imaging of the head showed a 2.5 × 1.3 cm hypointense shadow with clear borders in the left middle skull base and compression of the left temporal lobe. There was no ventricular pools enlargement or midline structures displacement. An arachnoid cyst was observed at the base of the left middle skull. After consultation with a neurosurgeon, no special treatment was suggested for the findings. Cerebrospinal fluid (CSF) protein levels and white blood cell counts were also examined. The D2R antibody titer detected by cell-based assays in the serum sample showed a ratio of 1:32, whereas the CSF sample showed negative findings. The other 15 autoimmune encephalitis antibodies, including anti-N-methyl-D-aspartate receptor (NMDAR) and anti-contactin-associated protein (CASPR), paraneoplastic antibodies, and oligoclonal bands, were negative in both samples. Thyroid ultrasound showed a nodule in the right lobe of the thyroid, which could be nodular goiter. Cardiac, abdominal, urological, and gynecological ultrasonography revealed no abnormalities. Chest computed tomography, electrocardiography, and electroencephalography did not show any abnormalities. The results of hormone examination showed a prolactin level of 13.8 μg/L and estradiol level of 107.4 pmol/L.

### Further diagnostic work-up

The Brief Psychiatric Rating Scale (BPRS) (5) score was 92, Hamilton Depression Scale (HAMD) score was 8 (6), Hamilton Anxiety Scale (HAMA) score was 9 (7), and Hypomania Checklist (HCL-32) score was 7 (8). This suggests that the patient had no obvious symptoms of depression, anxiety, or hypomanic episodes.

### Final diagnosis

The patient was diagnosed with schizophrenia according to Diagnostic and Statistical Manual of Mental Disorders (DSM-5) (9).

### Treatment

**Figure 1:**
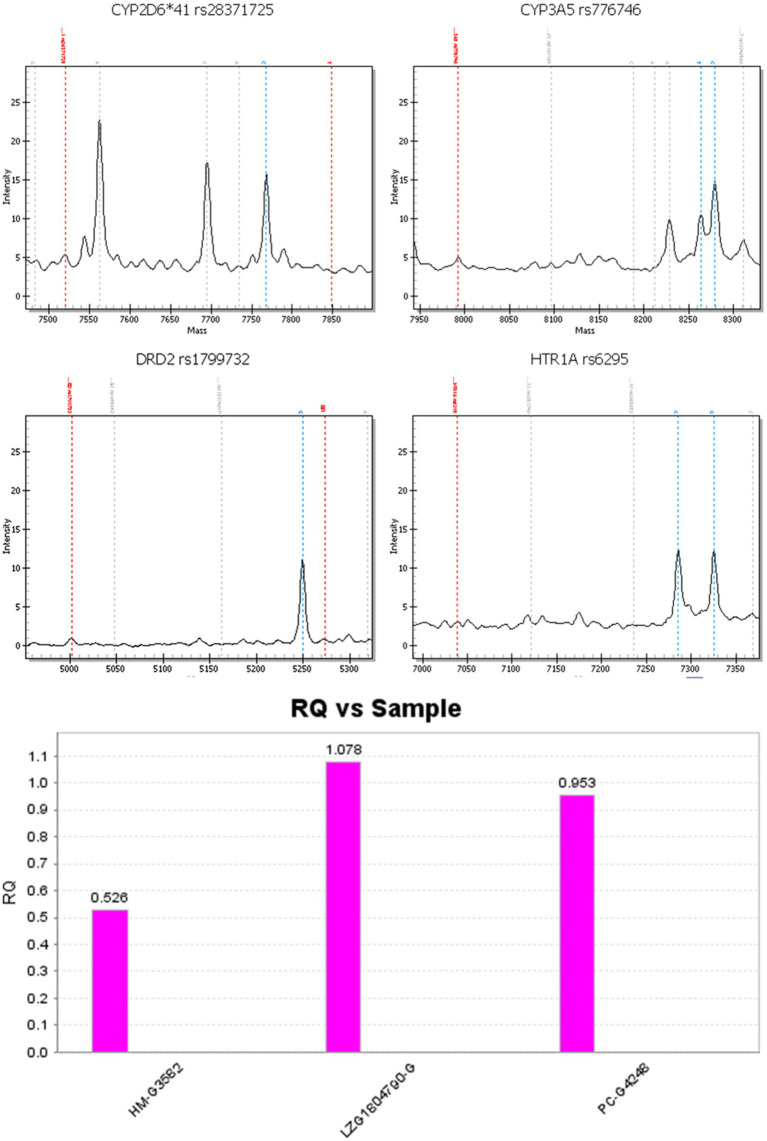
RQ vs. sample. Note: From left to right, the three samples are: CYP2D6*5 positive sample (gene deletion control), sample to be tested, CYP2D6*5 non-deletion sample (wild type control).

After diagnosis, the medications were gradually adjusted to a maximum dose of aripiprazole (15 mg twice daily) and olanzapine (10 mg twice daily) to control the psychiatric symptoms. Modified electric convulsive therapy was administered 10 times during hospitalization. However, the treatment efficacy was poor. At that time, the patient has been titrated to the maximum dosage of medications, yet the therapeutic response remains inadequate. Given this situation, it is imperative to refine the treatment plan. Consequently, genetic testing was considered indispensable for making a well-informed decision regarding the most suitable treatment drugs. Consequently, we proceeded directly with genetic testing to identify the appropriate treatment drugs. The antipsychotic drug metabolism gene test and *DRD2* gene test results suggested that the patient might response better to olanzapine, clozapine, risperidone, and aripiprazole; with a low risk of weight gain. Furthermore, the *HTR1A* gene test results suggested that she had a high chance of improvement of negative symptoms with several of these drugs. Patient DNA was genotyped using Mass ARRAY time-of-flight mass spectrometry (Sequenom Inc., United States) and real-time PCR (ABI VIIA7 real-time PCR Instrument, ABI Inc., United States). The gene test results are presented in Table 1 and Figure 1.

**Table 1:** Gene test results.

Test site	rs ID	cDNA change	Test results
CYP1A2*1F	rs762551	C>A	CA (heterozygous mutation)
CYP2D6*5		Gene deletion	Wild homozygote (not missing)
CYP2D6*10	rs1065852	100C>T	TT (homozygous mutation)
CYP2D6*41	rs28371725	2988G>A	GG (wild-type homozygous)
CYP3A5*3	rs776746	6986A>G	AG (homozygous mutation)
*DRD2*	rs1799732	-141C Ins/Del	Ins/Ins (wild-type homozygous)
*HTR1A*	rs6295	c.-1019G>C	GC (heterozygous mutation)

Olanzapine and clozapine are mainly metabolized and cleared by CYP1A2 metabolizing enzyme. The *CYP1A2* gene test results suggest that the patient was a CYP1A2 fast metabolizer with normal metabolic rate and normal sensitivity to olanzapine and clozapine. The dopamine receptor encoded by *DRD2* gene and pentraxin 1A receptor encoded by *HTRIA* gene are the targets of olanzapine and clozapine. Risperidone and aripiprazole are mainly metabolized and cleared by CYP2D6-metabolizing enzymes. The *CYP2D6* gene test results suggest that the patients was a CYP2D6 intermediate metabolizer, with reduced metabolism rate and increased sensitivity to risperidone and aripiprazole, and had an increased risk of neurological adverse effects, suggesting that the drug dose should be adjusted in conjunction with drug quasi-monitoring, and that the patients should be alerted to the occurrence of adverse drug reactions.

The active ingredient of paliperidone is an active metabolite of risperidone. Its pharmacological effect is similar to that of risperidone but it has a more direct effect. Considering the patient’s age and other factors, paliperidone extended-release tablets (3 mg in the morning and 9 mg in the evening) and olanzapine tablets (5 mg in the morning and 10 mg in the evening) were finally used to control psychiatric symptoms. After continuous drug treatment (Table 2), the patient’s psychiatric symptoms improved significantly, and her sleep improved. She had occasional verbal hallucinations and sense of insecurity and was discharged in October 2018. The drug plasma level examination results at the time of discharge indicated that the patient’s blood concentration of paliperidone was 48 ng/mL, which falls within the recommended treatment reference range outlined in the consensus guidelines for monitoring drugs used in neuropsychopharmacologic therapy, ranging from 20 to 60 ng/mL (10).

**Table 2:** Course of medication during hospitalization.

Days after admission	1	7	15	30	35	45 (discharge)
Drugs	Aripiprazole	Aripiprazole	Aripiprazole	Aripiprazole	Paliperidone extended-release tablets	Paliperidone extended-release tablets
	Olanzapine tablets	Olanzapine tablets	Olanzapine tablets	Olanzapine tablets	Aripiprazole	Aripiprazole
					Olanzapine tablets	Olanzapine tablets
Maximal dose, mg/d^a^	10	20	30	30	3	12
	2.5	7.5	15	20	15	0
					20	15

a Listed in the same vertical sequence as the drugs in the row above.

### Follow-up period and outcome

After being discharged from the hospital, the patient insisted on a monthly outpatient follow-up. During the patient’s continuous follow-up for more than 2 years, her treating physician found an interesting phenomenon. The patient’s symptoms fluctuated every time during her menstrual cycle, and the patient complained that she was “back to the state she was in before hospitalization.” She also had a significant weight gain, gaining 8 kg in approximately half a year after discharge. Her blood test results showed prolactin level of 34.6 μg/L (normal range 4–23 μg/L). The patient complained that she had been taking her medication as prescribed and denied any increase or decrease in the dosage or missed doses. To quantify these symptoms, in addition to blood monitoring for hormone levels (prolactin and estradiol) (Table 3), we used the Positive and Negative Syndrome Scale (PANSS) to assess the severity of the patient’s symptoms at each outpatient follow-up visit. Throughout the treatment period, paliperidone (6 mg twice daily) was used as the main medication in combination with clozapine (50 mg twice daily). The treating physician adjusted the type and dose of medication according to the patient’s symptoms; however, a stable effect was not achieved, and the symptoms fluctuated periodically with the menstrual cycle. The main medications used during the follow-up period after the patient’s discharge from the hospital, with each medication adjusted as a treatment regimen are presented in Tables 4–6.

**Table 3:** Blood test results of prolactin and estradiol for six consecutive months.^a^

Month		1	2	3	4	5	6
PRL (μg/L)		32.7	34.9	36.5	32.8	33.7	34.2
E_2_ (pmol/L)	The third day after menstruation	114.9	110.7	112.6	110.4	111.8	114.3
	Menstrual period	69.6	72.8	79.4	70.9	67.6	74.5

APRL, prolactin; E_2_, estradiol.

a After informed consent was obtained from the patient and her family, the hormone levels were monitored on the first and third days after menarche for 6 months.

**Table 4:** Antipsychotic treatment between 2018 and 2019.

Sequence of therapeutic schedule	1	2	3	4	5
Drugs	Paliperidone extended-release tablets	Paliperidone extended-release tablets	Paliperidone extended-release tablets	Paliperidone extended-release tablets	Paliperidone extended-release tablets
	Olanzapine	Olanzapine	Olanzapine	Aripiprazole	Olanzapine
		Aripiprazole Tablets	Aripiprazole		Aripiprazole
Maximal dose, mg/d^a^	12	12	9	12	12
	15	20	10	0	20
		5	5		10

a Listed in the same vertical sequence as the drugs in the row above.

**Table 5:** Antipsychotic treatment between 2020 and 2021.

Sequence of therapeutic schedule	6	7	8	9	10
Drugs	Paliperidone extended-release tablets	Paliperidone extended-release tablets	Paliperidone extended-release tablets	Paliperidone extended-release tablets	Paliperidone palmitate injection
	Clozapine	Clozapine	Clozapine	Clozapine	Clozapine
				Paliperidone palmitate injection	
Maximal dose, mg/d^a^	12	9	9	3	150 (per month)
	100	100	100	100	100
				150 (per month)	

a Listed in the same vertical sequence as the drugs in the row above.

**Table 6:** Antipsychotic treatment from August 2021 to 2022.

Sequence of therapeutic schedule	11	12	13
Drugs	Paliperidone palmitate injection	Paliperidone palmitate injection	Paliperidone palmitate injection
	Aripiprazole	Aripiprazole	Aripiprazole
	Clozapine	Clozapine	Clozapine
Maximal dose, mg/d^a^	100 (per month)	100 (per month)	100 (per month)
	10	5	5
	50	50	25

a Listed in the same vertical sequence as the drugs in the row above.

The patient, an adolescent female diagnosed with schizophrenia, experienced worsening psychiatric symptoms during each menstrual period. The blood concentration of paliperidone ranged from 12 to 18 ng/mL when her menstrual cycle began. However, outside of her menstrual period, the plasma level of paliperidone ranged from 30 to 55 ng/mL. The patient was currently receiving the maximum therapeutic dose of paliperidone as approved by the medication’s label, and the treating physician opted not to further increase the dosage. There were several reasons for this decision. Firstly, the patient and her parents were reluctant to exceed the maximum dose specified on the label. Additionally, the attending physician believed that even with an escalation of paliperidone dosage, achieving the desired plasma concentration during the patient’s menstrual cycle might remain challenging.

Finally, the patient had been taking antipsychotic drugs for a long time, which led to elevated prolactin and significant weight gain. These factors, along with the stigma of adhering to medication during school, may have contributed to the patient’s poor medication adherence. On a follow-up visit in August 2020, her attending physician attempted to adjust her treatment after communication with the patient and her family, and after obtaining informed consent, replaced oral paliperidone with a chemically identical, longer-lasting, and more stable long-acting injection of paliperidone palmitate. The starting dose was 150 mg, and 1 week later, the injection was administered intramuscularly at a monthly dose of 100 mg. After switching to the injectable preparation, the patient’s condition was stable, and so far, no further psychiatric symptoms have occurred for more than 2 years, either at ordinary times or during the menstrual cycle. The blood concentration of paliperidone ranged from 25 to 53 ng/mL. Currently, the patient’s condition is well controlled.

**Figure 2:**
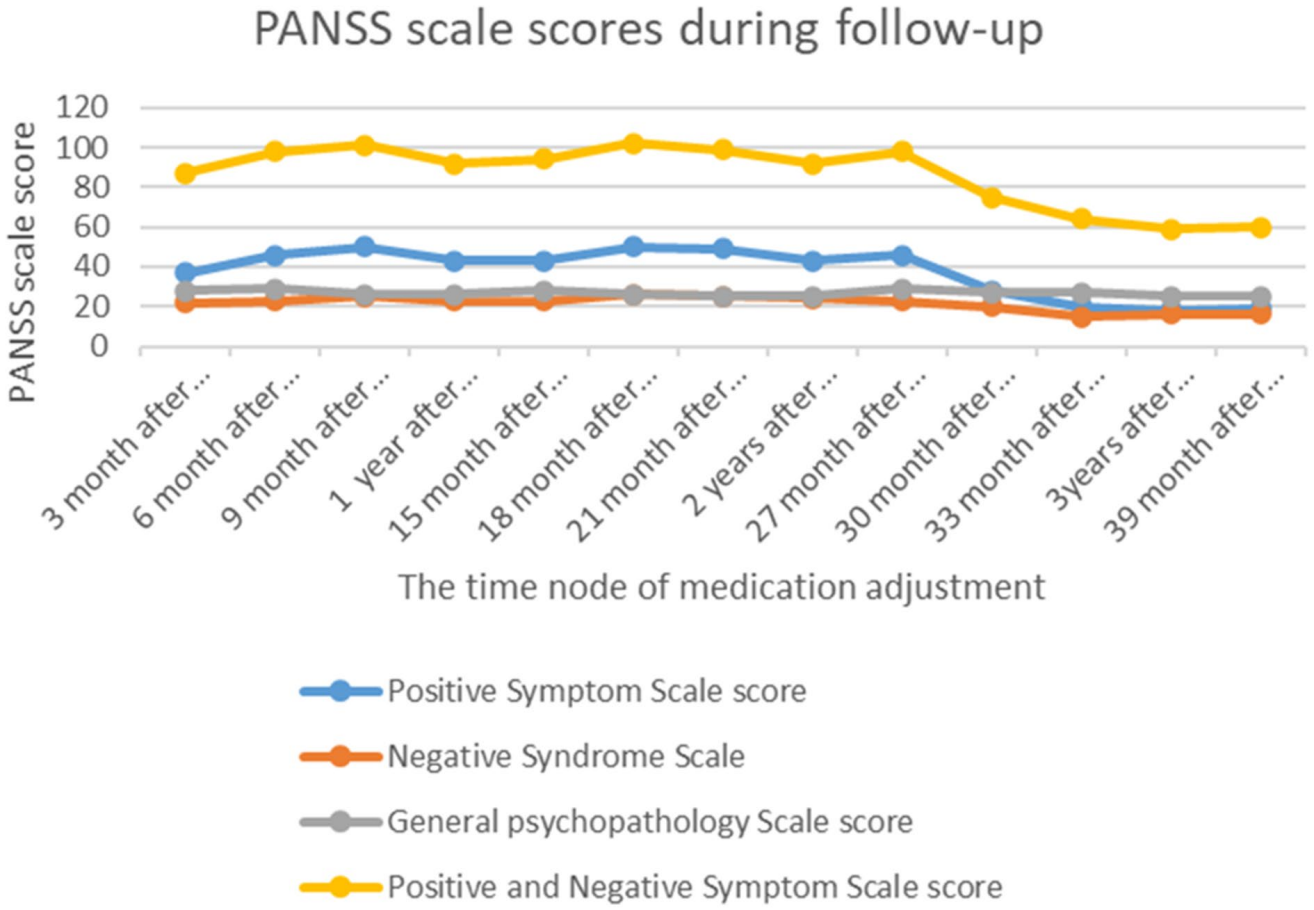
PANSS scale score trend chart during follow-up. Note: The patient was followed up every 3 months after discharge, and the attending clinician adjusted the medication according to the patient’ chief complaints and symptoms and evaluated her symptoms using the Positive and Negative Syndrome Scale.

The trend of the patient’s scores on the corresponding PANSS scale over the course of more than 3 years of follow-up is shown in Figure 2. The scores decreased significantly after the medication was adjusted to paliperidone injectable injection (30 months after discharge), indicating that the patient’s overall psychiatric symptoms were significantly controlled.

## Discussion

In the present case, the patient’s medical history revealed obvious hallucinations, delusions, incoherent speech, off-topic answers, tension, withdrawal behavior, decreased emotional expression, and a lack of motivation. After experiencing the symptoms, the patient showed a significant decline in academic performance, interpersonal communication, and other aspects of functioning that lasted for 6 months. The overall symptoms met the diagnostic criteria for schizophrenia according to DSM-5. The patient was a young woman with a slow onset and long course of disease. The examination results showed normal thyroid function; thus, eliminating the diagnosis of mental disorders caused by thyroid disease. CSF examination ruled out autoimmune encephalitis. The patient denied substance abuse and had a negative urinalysis result, which eliminated the possibility of a psychiatric disorder caused by psychoactive substances. Previously administered antipsychotics (paliperidone modified-release tablets and olanzapine) were effective; however, fluctuations in psychiatric symptoms, such as recurrent auditory hallucinations, increased delusions, and increased alertness, were observed. These symptoms were similar to those reported previously and the patient did not report any new symptoms. Almost all symptom fluctuations were related to the menstrual cycle. The patient and her family members reported that she had been taking the medications as prescribed and she had not taken any other medication on her own that could have caused changes in blood levels.

Numerous studies have shown that the physiological cycle can cause fluctuations in psychiatric symptoms in patients with schizophrenia (4, 11–13). Estrogen and prolactin are the two major hormones associated with these fluctuations. Estrogen has a protective effect against psychosis, which worsens when estrogen levels are low during the pre- and post-menstrual cycles (14). Estrogen levels vary during different stages of the physiological cycle (11). Psychiatric symptoms may appear or worsen during the declining phase of the menstrual cycle, that is the first week after the start of menstruation, when the corpus luteum shrinks and the blood levels of progesterone and estrogen are low. However, during the ovulatory and luteal phases, estrogen levels are higher, and patients may experience fewer psychiatric symptoms. Estrogen has an antagonistic effect on dopamine and 5 HT and can change the mental traits of patients by regulating the release and metabolism of dopamine and 5 HT (15). When estrogen levels are low during menstruation, the antagonistic effect of estrogen on dopamine and 5 HT is weakened, which may lead to the worsening of psychotic symptoms (12).

Furthermore, changes in prolactin levels are associated with symptom fluctuations in patients with schizophrenia (16, 17). In this case, the patient’s hormone levels were within the normal range before treatment; however, after a period of drug treatment, the prolactin level was observed to be significantly higher than normal, which also verified that antipsychotic drugs could cause hyperprolactinemia (18). Prolactin affects the function of the anterior pituitary gland and hypothalamus. Studies have shown that patients with high prolactin levels are less responsive to treatment (19). Higher prolactin levels are associated with more severe positive (20) and negative (21) psychotic symptoms in patients with uncontrolled symptoms. This finding is consistent with the patient’s performance in this case.

Alternatively, changes in a female’s menstrual cycle may affect the pharmacokinetics of a drug, thereby affecting the blood concentration of oral or injected drugs. At different stages of the menstrual cycle, female patients may experience changes in the efficacy or side effects of medications. This may be related to the effect of estrogen on the liver enzyme system, which is one of the main sites for drug metabolism (22). Most antipsychotic drugs are metabolized by CYP1A2, CYP2C19, CYP2D6, and/or CYP3A4 (10). Both estrogen and progesterone are substrates of CYP1A2, and have an inhibitory effect on this enzyme (23). This may result in high drug plasma concentrations in females when the hormone levels are high (e.g., premenopausal) and relatively low concentrations when the hormone levels are low (e.g., postmenopausal). This may affect the action of drugs, including clozapine and olanzapine (24).

Risperidone and paliperidone are mainly metabolized by CYP2D6 (22). In the present case, the patient was an intermediate metabolizer of CYP2D6, with a low metabolic rate and high sensitivity to risperidone and aripiprazole. Such patient may have higher enzyme activity, thereby accelerating the metabolism and excretion of drugs and leading to a decrease in blood drug concentration. When the level of estrogen in the body decreases, the synthesis of CYP2D6 is reduced, thus slowing down the metabolic rate of oral drugs and impairing their efficacy. In our patient, the blood concentration of paliperidone was below the normal range when her menstrual cycle began but remained within the normal range outside of her menstrual period. An increase in the blood drug concentration also makes the side effects of drugs relatively obvious, causing discomfort to patients (24). Therefore, when the efficacy of a previously effective antipsychotic drug dose is reduced during the estrogen decline phase, increasing the dose may not a treatment option because it may increase the risk of adverse effects. Thus, eliminating the first metabolic pathway by changing the drug type or delivery method will ensure improvement in the therapeutic effect in such cases.

For long-acting injections, the drug release rate is relatively stable, and drug metabolism is not affected by the patient’s sex (25, 26); thus, the blood drug concentration is relatively less affected by the menstrual cycle and hormones. In contrast, metabolism of oral drugs is more complex and may be influenced by the menstrual cycle. Estrogen has a greater effect on the blood concentration of oral psychiatric drugs than that of long-acting injectable drugs during the menstrual cycle.

In the clinical treatment of schizophrenia, continuous adjustment of drug types and doses and optimization of medication strategies are important. Ensuring drug efficacy, conducting early and timely intervention, reducing corresponding adverse reactions, and improving the treatment compliance of patients are common challenges faced by clinicians. The general clinical course of treatment using atypical antipsychotics involves increasing the dose or combining atypical antipsychotics with other drugs. In this case, medication adjustments were also made for 3 years, but with poor results. To rule out the effect of changes in blood drug concentration during the menstrual cycle and a decrease in patient medication compliance, the attending physician decided to change the form of medication without changing the type. Therefore, the oral form was changed to a long-acting injection, which ensured that the drug concentration in the patient’s body remained within the therapeutic range. The long-acting injection is not only superior to oral dosage forms in improving patient compliance and preventing recurrence (27, 28) but can improve patients’ social function earlier and for a longer duration (29). The treatment results of the patient confirmed that the long-acting injection provided a durable and stable improvement in psychiatric symptoms.

Therapeutic drug monitoring (TDM) and pharmacogenomic testing are indispensable tools in common clinical practice. TDM allows healthcare professionals to fine-tune medication dosages, ensuring that patients receive the optimal amount of a drug to achieve the desired therapeutic effect while minimizing side effects. On the other hand, pharmacogenomic testing takes personalized medicine to the next level by analyzing an individual’s genetic makeup to predict their response to specific medications. It aids in selecting the most appropriate drugs and dosages based on genetic factors, reducing the risk of adverse reactions and improving patient safety. Together, TDM and pharmacogenomics help deliver more effective, safer, and patient-centered healthcare. In our patient, during the onset of her menstrual cycle, the blood concentration of paliperidone fell below the established therapeutic range. However, outside of her menstrual period, the paliperidone concentration consistently remained within the therapeutic range. The results of genetic monitoring showed that paliperidone, an intermediate metabolizer, is safe and effective for patients, and switching to a different formulation of the same drug, rather than increasing doses or introducing drug combinations, may be a solution to fluctuations in symptoms during specific periods. In clinical practice, TDM and genetic testing should be performed in a timely manner to identify effective drugs for targeted treatment.

## Conclusion

For female patients with schizophrenia with effective drug treatment, if the mental symptoms fluctuate periodically with the physiological cycle, and the effect of drug adjustment is not satisfactory, we recommend replacement with a similar long-acting injectable agent. Based on this case, we suggest that changes in hormone levels should be monitored in female adolescents during treatment and other forms of drugs that are less affected by hormones, such as long-acting injections should be administered. This may help to achieve the desired therapeutic effect. However, further high-quality research is required to promote the development of such formulations.

## Data availability statement

The original contributions presented in the study are included in the article/supplementary material, further inquiries can be directed to the corresponding author.

## Ethics statement

Written informed consent was obtained from the individual(s), and minor(s)’ legal guardian/next of kin, for the publication of any potentially identifiable images or data included in this article.

## Author contributions

FW: Project administration, Writing – review & editing, Investigation, Conceptualization, Data curation, Writing – original draft. JC: Writing – original draft, Resources, Supervision, Writing – review & editing. LG: Writing – review & editing, Data curation, Formal analysis, Software. ZgL: Data curation, Formal analysis, Software, Writing – review & editing. ZeL: Conceptualization, Funding acquisition, Investigation, Methodology, Project administration, Resources, Supervision, Writing – review & editing.
